# Targeted mutagenesis in ryegrass (*Lolium* spp.) using the CRISPR/Cas9 system

**DOI:** 10.1111/pbi.13359

**Published:** 2020-03-17

**Authors:** Yunwei Zhang, Yidong Ran, Istvan Nagy, Ingo Lenk, Jin‐Long Qiu, Torben Asp, Christian S. Jensen, Caixia Gao

**Affiliations:** ^1^ State Key Laboratory of Plant Genomics Institute of Microbiology Chinese Academy of Sciences Beijing China; ^2^ Genovo Biotechnology Co. Ltd Tianjin China; ^3^ Department of Molecular Biology and Genetics, Crop Genetics and Biotechnology Aarhus University Slagelse Denmark; ^4^ Research Division DLF Seeds A/S Store Heddinge Denmark; ^5^ State Key Laboratory of Plant Cell and Chromosome Engineering Center for Genome Editing Institute of Genetics and Developmental Biology Innovation Academy for Seed Design Chinese Academy of Sciences Beijing China

**Keywords:** ryegrass, CRISPR/Cas9, DMC1

Ryegrass is one of the most important forage crops worldwide. It is the basis for 80% of milk production and 70% of meat production and has major economic importance. Breeding programmes for ryegrass started in the 1920s, and breeders have mainly relied on repeated phenotypic and recently genotypic selection of elite individuals. Although this approach has led to significant improvements in several characters including rust resistance, spring growth and aftermath heading, it tends to be laborious, expensive and time‐consuming, mainly due to gametophyte self‐incompatibility in most ryegrass species (Sampoux *et al.*, 2011). In order to overcome some of the limitations of traditional introgression and selective breeding, modern methods of mutation induction offer attractive opportunities to target specific genes of interest and directly introduce allelic variability. In the last decade, the Clustered Regularly Interspaced Short Palindromic Repeats/CRISPR‐associated endonuclease 9 (CRISPR/Cas9) system has been extensively used in most crops and is paving the way to precision trait improvements in factors including yield, quality, biotic‐ and abiotic stress resistance and breeding rate (Chen *et al.*, 2019; Ran *et al.*, 2017; Wang *et al.*, 2014). However, this powerful tool for genome editing has not yet been used in ryegrass. Meiosis arose early during the evolution of eukaryotes and is vital for sexual reproduction, not only in relation to genomic stability but also to genetic diversity. Meiotic studies of plants in the areas of crop fertility and genetic variation have important potential agronomical applications. *DMC1 (DISRUPTED MEIOTIC cDNA1),* initially identified in yeast (Bishop *et al.*, 1992) as a homolog of the bacterial strand exchange protein RecA, is a crucial meiotic recombinase. Here, we describe the use of the CRISPR/Cas9 system to introduce mutations in *LpDMC1* in two species: Italian ryegrass (*Lolium perenne* ssp. *multiflorum*) and perennial ryegrass (*Lolium perenne*). We succeeded in obtaining both T0 homozygous and heterozygous mutants, and the T0 null mutants of Italian ryegrass exhibited complete male sterility and severely disordered meiosis with univalents and multivalents appearing at diakinesis.

To see whether mutations could be introduced into ryegrass using the CRISPR/Cas9 system, we generated a sgRNA (*TS*‐*LpDMC1)* targeting exon 5 of *LpDMC1,* with an *Xce*I restriction enzyme site near the protospacer‐adjacent motif (PAM) for ease of analysis (Figure 1a). Because plant tissue culture and genetic transformation are time‐consuming, we tested the activities of sgRNA in a protoplast transient expression system as described by Shan *et al. *(2013). *TS*‐*LpDMC1* promoted by TaU6 was co‐introduced with SpCas9 into ryegrass protoplasts by PEG‐mediated transformation. After 40‐ to 48‐h incubation, analysis of genomic DNA using a PCR restriction enzyme digestion assay (PCR/RE) demonstrated the occurrence of indel mutations at the target site (Data not shown).

**Figure 1:**
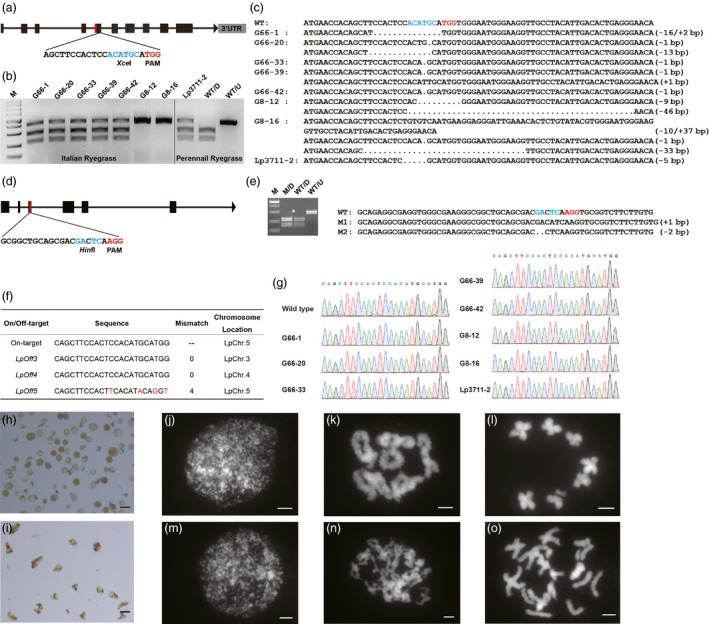
Targeted mutagenesis in ryegrass using the CRISPR/Cas9 system. (a) Region of *LpDMC1* exon 5 targeted by the CRISPR/Cas9 system. The PAM is in red, and the restriction enzyme site within the targeted region is in blue. (b) Outcomes of PCR/RE assays to detect CRISPR/Cas9‐induced mutations in T0 individuals of the two species. G66 and G8 represented the Italian ryegrass cultivar Gepetto, and Lp3711 represented the perennial ryegrass cultivar Goyave. Lanes labelled WT/D and WT/U indicate the wild type with and without *Xce*I digestion. (c) Sequence alignments of WT and mutants amplicons (‘−’ as deletions and ‘+’ as insertions). (d) Region of *LpCENH3* exon 3 targeted by CRISPR/Cas9. (e) Results of PCR/RE assays and sequencing of *LpCENH3* mutations induced by CRISPR/Cas9 in ryegrass protoplasts. The white star indicates mutant PCR products. (f) Predicted potential *TS‐LpDMC1* off‐target sites. (g) Sanger sequencing results of PCR products harbouring *LpOff3* and *LpOff4* in the 8 *Lpdmc1* mutants. (h) and (i) Wild‐type and *Lpdmc1* pollen grains stained with 1% I_2_‐KI solution. Bars = 50 μm. (j–l) Meiosis in the wild type. (m–o) Meiosis in a *Lpdmc1* mutant. (j) and (m) leptotene, (k) and (n) diplotene, (l) and (o) diakinesis. Bars = 5 μm.

To determine whether the CRISPR/Cas9 method was applicable to other ryegrass genes, we targeted the ryegrass orthologue of centromere‐specific histone H3 variant (CENH3). In *Arabidopsis thaliana*, CENH3 ensures faithful transmission of the genome at cell division, and when *cenh3* null mutants producing altered CENH3 proteins are crossed with wild type, many haploid *Arabidopsis* plants are generated (Ravi and Chan, 2010). When we co‐transformed a sgRNA targeting exon 3 of *LpCENH3* along with SpCas9 into protoplasts (Figure 1d), PCR/RE analysis revealed frameshift mutations at the target site (Figure 1e). These results show that CRISPR/Cas9 can be used to generate mutations in ryegrass.

Next, the sgRNA expression cassette was combined with SpCas9 in a single DNA construct by GIBSON Assembly and introduced along with a hygromycin‐resistant plasmid into preconditioned embryogenic callus (EC) lines of ryegrass by gold particles bombardment. To generate these EC lines, seeds of Italian ryegrass cultivar Gepetto and perennial ryegrass cultivar Goyave were de‐husked and sterilized, and somatic EC lines were established as described (Ran *et al.*, 2014). Three separate lines designated Gepetto‐8, Gepetto‐66 and Goyave LMG LDF‐Lp3711 (provided by DLF Seeds) with outstanding regeneration ability were selected for transformation. After bombardment, the EC was transferred to hygromycin medium. Surviving calli were obtained after 4 weeks’ induction and sub‐culture. Thereafter, they were regenerated for 8 weeks; and plantlets with established roots were transferred to potting mix for mutants’ identification. The mean transformation efficiencies of these three lines were 3.83%, 4.50% and 2.66%, respectively. The entire experimental cycle took approximately 10 months from target design to mutant identification. Compared with conventional methods, CRISPR/Cas9 provides a rapid and straightforward method for genetic manipulation of ryegrass.

T0 generation *LpDMC1* knockout mutants were identified by PCR/RE using the same primers as in the protoplast assays (Figure 1b), followed by Sanger sequencing. In total, we obtained eight mutants in two species: seven of Italian ryegrass and one of perennial ryegrass. G8‐12 was a homozygous mutant with a 9 bp in‐frame deletion in one allele. The other non‐wild‐type plant G8‐16 was a mosaic, with three mutations: −1 bp, −33 bp deletions and −10/+37 bp deletion/insertion. The other six were heterozygotes (Figure 1c). The genome editing efficiencies of *LpDMC1* in Gepetto‐8, Gepetto‐66 and Goyave LMG LDF‐Lp3711 were 11.63%, 11.11% and 5.88%, respectively.

To detect off‐target events, putative off‐target sites of *TS*‐*LpDMC1* were predicted using the draft genome sequence of the forage grass *Lolium perenne* (Byrne *et al.*, 2015) (Figure 1f). Two non‐mismatch off‐target sites, *LpOff3* and *LpOff4,* with highly similar surrounding sequences, which could produce mutations in two different non‐coding regions, were selected for further study. PCR products that included *LpOff3*/*LpOff4* from the eight mutants were sequenced, but no mutations were detected (Figure 1g). The next‐closest match was *LpOff5*, which had an imperfect PAM, suggesting that off‐target events would be unlikely (Figure 1f).

Because Italian and perennial ryegrass are both gametophyte self‐incompatibility and have different vernalization requirements, it was difficult and time‐consuming to obtain homozygous T1 mutants. Since disruption of *DMC1* in *Arabidopsis*, rice and barley usually leads to reduced fertility, we examined the pollen grains produced by wild type and the T0 *Lpdmc1* mutant G8‐16. Upon iodine‐potassium iodide (I_2_‐KI) staining, the pollen grains from the wild type appeared mostly round, while those from G8‐16 were empty and shrunken (Figure 1h,i). This phenotype points to complete male sterility, as in the *Osdmc1a Osdmc1b Tos‐17* double insertion mutant of rice (Wang *et al.*, 2016). The wild type pollen grains did not stain as strongly as those of rice, probably due to the difference in starch content between Italian ryegrass and rice. To clarify what happened during *Lpdmc1* meiosis, we stained meiotic chromosomes with 4’,6‐diamidino‐2‐phenylindole (DAPI). The meiotic chromosomes of the *Lpdmc1* mutant behaved normally at leptotene (Figure 1j,m). However at diplotene, whereas circular or figure‐of‐eight shapes of the chromatids were observed in wild type (Figure 1k), in the *Lpdmc1* mutant the chromatids were stuck together, mostly due to the existence of multivalents (Figure 1n). At diakinesis, unlike the wild type, which formed 7 bivalents (Figure 1l), condensed univalents and multivalents were scattered throughout the mutant nuclei (Figure 1o). These results point to a meiotic defect in the *Lpdmc1* mutant.

In summary, we were able to introduce mutations in two genes, *LpDMC1* and *LpCENH3,* of ryegrass *in vivo* using the CRISPR/Cas9 system*.* We obtained eight T0 knockout mutants of *LpDMC1* in Italian and perennial ryegrass. The *Lpdmc1* null mutants were completely male sterile with severely disrupted meiosis, indicating that DMC1, a highly conserved protein, plays a pivotal role in meiosis in many species. We are now using genome editing tools to improve other agronomic traits in ryegrass. We anticipate that this method will come to be used routinely in this economically important forage crop.

## Conflicts of interest

The authors declare no conflict of interest.

## Author contributions

Y.Z., J.‐L.Q., C.S.J. and C.G. designed the experiments; I.N. and T.A. provided genomics and bioinformatics support; Y.R. performed the genetic transformation of ryegrasses; I.L. and C.S.J. provided the ryegrass seeds; Y.Z. performed the molecular and cytologic experiments; C.G supervised the project; and Y.Z., Y.R. and C.G. wrote the manuscript.
